# Unraveling the Complexity of Non-typhoidal Salmonella Genitourinary Infection in HIV: A Case Report and Management Strategy

**DOI:** 10.7759/cureus.79739

**Published:** 2025-02-27

**Authors:** Piyush Puri, Daniel Miller, Ramsha Durrani, Santino E Patrizi, Nadya Chowdhury

**Affiliations:** 1 Internal Medicine, Icahn School of Medicine at Mount Sinai, Queens Hospital Center, New York, USA

**Keywords:** hiv aids, non-typhoidal salmonellae, recurrent uti, salmonella infection, uti

## Abstract

*Salmonella* urinary tract infections (UTIs) are rare and typically affect immunocompromised or anatomically predisposed individuals. While usually foodborne, transmission can also occur from pets or person to person. Symptoms mimic common UTIs and may present with or without prior gastrointestinal illness. We report the case of a 56-year-old HIV-positive male with poor medication compliance who presented with altered mental status and burning micturition. Urine and blood cultures revealed non-typhoidal *Salmonella*, prompting targeted antibiotic therapy. Treatment involves at least 14 days of antibiotics, with repeat urine cultures to ensure eradication. Rising antibiotic resistance necessitates awareness of local sensitivity patterns.

## Introduction

Non-typhoidal *Salmonella* (NTS) infections are a significant concern for individuals with HIV, particularly in cases of severe immunosuppression. While *Salmonella* typically affects the gastrointestinal tract, extraintestinal manifestations, including urinary tract infections (UTIs), can occur in immunocompromised patients. These infections are rare but can lead to serious complications such as bacteremia and urosepsis [1,2].

HIV-infected patients have a 20 to 100-fold increased risk of acquiring *Salmonella* infections compared to the general population. The presence of NTS in the urinary tract often indicates an underlying predisposing factor, with HIV being a significant risk factor [1,2]. This case report aims to highlight the unique presentation, diagnostic challenges, and management of NTS UTI in an HIV-positive patient. While salmonellosis can sometimes be an early indicator of HIV, it primarily affects those with severe immunosuppression, particularly with CD4 counts below 100/mm³. The mortality rate is high at 50%, comparable to the 52.6% seen in other immunosuppressed patients [3].

By presenting this case, we seek to raise awareness among clinicians about the importance of considering NTS as a potential causative agent in HIV patients presenting with urinary symptoms. Furthermore, this report underscores the need for prompt diagnosis and appropriate treatment to prevent complications and improve patient outcomes in this vulnerable population [1,3].

## Case presentation

A 56-year-old male, with a past medical history significant for HIV (with documented non-compliance with Biktarvy), seizure disorder (also with poor medication compliance), and homelessness, presented to the emergency department with altered mental status and urinary incontinence.

The patient was found outside a fire department station, noted to have soiled himself, and been altered. On examination, the patient could not provide much history but stated he did not regularly take his medications. He reported being lost with mild shortness of breath and chest tightness. At baseline, the patient was homeless and able to perform all activities of daily living independently. Similar presentations had occurred three times in the preceding two weeks.

Complete blood count showed increased white blood cell count and neutrophilia (Table 1). Urine analysis was positive for UTI (Table 2). Urine and blood cultures came back positive for non-enteric *Salmonella* (Table 3).

**Table 1:** Complete blood count.

Component	Results	Reference range and units
White blood cells	15.01	4.80–10.80 × 10³/µL
Red blood cells	4.09	4.70–6.10 × 10⁶/µL
Hemoglobin	11.5	14.0–18.0 g/dL
Hematocit	36.6	42.0–52.0%
Mean corpuscular volume	89.5	80.0–99.0 fL
Mean corpuscular hemoglobin	28.1	27.0–31.0 pg
Mean corpuscular hemoglobin concentration	31.4	29.8–35.2 g/dL
Mean platelet volume	10.0	8.7–12.9 fL
Red blood cell distribution width	16.0	12.0–15.0%
Platelet	502	150–450 × 10³/µL
Neutrophil %	93.7	44.0–70.0%
Lymphocyte %	2.3	20.0–45.0%
Monocyte %	3.1	2.0–10.0%
Eosinophil %	0.0	1.0–4.0%
Basophil %	0.1	0.0–2.0%
Immature granulocytes %	0.8	0.0–2.0%
Neutrophil absolute	14.07	2.10–7.60 × 10³/µL
Lymphocyte absolute	0.34	1.00–4.90 × 10³/µL
Monocyte absolute	0.47	0.10–1.10 × 10³/µL
Eosinophil absolute	0.00	0.10–0.40 × 10³/µL
Basophil absolute	0.01	0.00–0.20 × 10³/µL
Immature granulocytes absolute	0.12	0.00–0.20 × 10³/µL
Nucleated red blood cells absolute	0.00	*≤*0.00 × 10³/µL

**Table 2:** Urine analysis.

Component	Test results	Reference range and units
pH	6.0	5.0–8.0
Color	Yellow	Yellow
Appearance	Turbid	Clear
Glucose qualitative	Negative	Negative, mg/dL
Bilirubin	Negative	Negative
Ketones	Negative	Negative, mg/dL
Specific gravity	1.013	1.005–1.030
Blood	Large	Negative
Protein	100	Negative, mg/dL
Urobilinogen	0.2	0.2–1.0 EU/dL
Nitrite	Positive	Negative
Leukocyte esterase	Large	Negative
White blood cells	2,142.6	0.0–5.0/HPF
Red blood cells	35.0	0.0–5.0/HPF
Bacteria	Moderate	None seen, /HPF
Squamous epithelial cells	0–5	0–5/HPF
Cast	3–5	0–2/LPF

**Table 3:** Urine culture and blood culture.

Test	Result
Urinalysis	Positive for urinary tract infection
Urine culture	>100,000 CFU/mL. *Salmonella* species, not typhi/paratyphi, sensitive to ampicillin, ciprofloxacin, and trimethoprim/sulfamethoxazole; resistant to ceftriaxone.
Blood cultures (aerobic)	*Salmonella *species, not typhi/paratyphi, Gram-negative rods

The patient received intravenous fluids and was initiated on cefepime and levetiracetam. After cultures, Infectious Disease was consulted, cefepime was discontinued, and the patient was started on oral trimethoprim/sulfamethoxazole (800-160 mg) twice daily. His home medications for seizure disorder were resumed. A four-week course was planned, considering his HIV status. Coordination with virology services was initiated for follow-up and management of his HIV.

## Discussion

*Salmonella* is increasingly recognized as a leading cause of foodborne illness, primarily linked to contaminated chicken eggs [4,5]. While extraintestinal focal infections have been reported, UTIs caused by NTS remain rare. Globally, the incidence of NTS-positive urine cultures ranges from 0.015% to 0.118%, with approximately 18% of affected patients becoming chronic urinary carriers [6]. NTS-related UTIs are typically associated with immunosuppression, chronic conditions such as diabetes, or structural genitourinary abnormalities [7].

NTS UTI often indicates underlying risk factors, such as undiagnosed immunosuppression or genitourinary abnormalities [7]. Literature suggests its pathogenesis occurs through direct urethral invasion or hematogenous spread following gastroenteritis, though our patient had no prior gastrointestinal symptoms [7]. Antibiotic treatment duration varies from two weeks for mild cases to over six weeks [8]. Gorelik et al. found that NTS UTIs were not significantly associated with age, sex, diabetes, immunosuppression, or urologic abnormalities. However, a notable link was observed between NTS UTI and underlying urologic malignancies (23.5% vs. 0%, p = 0.023). Given the small sample size in the Gorelik et al. study, the findings should be interpreted cautiously, especially as no effect of age or immunity on NTS infection was observed.

A 14-day course of appropriate antibiotics, or a longer duration based on the organism’s sensitivity pattern, is effective in treating *Salmonella* UTIs. Given rising antibiotic resistance, clinicians must consider local sensitivity trends. Repeat urine cultures are essential to confirm eradication of the infection [9,10].

## Conclusions

NTS UTIs are more common in immunocompromised individuals, particularly those with HIV, and may require prolonged antibiotic treatment due to complications and recurrence. A distinct subgroup of patients with NTS UTI, but without gastroenteritis, shows an association with urologic malignancies. Atypical presentations, such as orchitis/epididymitis, can occur, making diagnosis and management challenging. Given the potential for severe illness in immunocompromised patients, clinicians should be aware of the varied presentations of salmonellosis to ensure timely diagnosis and treatment.
